# High tumor cell platelet‐derived growth factor receptor beta expression is associated with shorter survival in malignant pleural epithelioid mesothelioma

**DOI:** 10.1002/cjp2.218

**Published:** 2021-05-06

**Authors:** Hely Ollila, Juuso Paajanen, Henrik Wolff, Ilkka Ilonen, Eva Sutinen, Katja Välimäki, Arne Östman, Sisko Anttila, Eeva Kettunen, Jari Räsänen, Olli Kallioniemi, Marjukka Myllärniemi, Mikko I Mäyränpää, Teijo Pellinen

**Affiliations:** ^1^ Institute for Molecular Medicine Finland (FIMM), Helsinki Institute of Life Science (HiLIFE) University of Helsinki Helsinki Finland; ^2^ Individualized Drug Therapy Research Program, Faculty of Medicine University of Helsinki Helsinki Finland; ^3^ Department of Pulmonary Medicine Heart and Lung Center, University of Helsinki and Helsinki University Hospital Helsinki Finland; ^4^ Laboratory of Pathology Finnish Institute of Occupational Health Helsinki Finland; ^5^ Department of Pathology University of Helsinki and Helsinki University Hospital Helsinki Finland; ^6^ Department of General Thoracic and Esophageal Surgery Heart and Lung Center, University of Helsinki and Helsinki University Hospital Helsinki Finland; ^7^ Department of Oncology‐Pathology Karolinska Institutet Solna Sweden

**Keywords:** mesothelioma, prognosis, platelet‐derived growth factor receptor beta, fibroblast

## Abstract

Malignant pleural mesothelioma (MPM) has a rich stromal component containing mesenchymal fibroblasts. However, the properties and interplay of MPM tumor cells and their surrounding stromal fibroblasts are poorly characterized. Our objective was to spatially profile known mesenchymal markers in both tumor cells and associated fibroblasts and correlate their expression with patient survival. The primary study cohort consisted of 74 MPM patients, including 16 patients who survived at least 60 months. We analyzed location‐specific tissue expression of seven fibroblast markers in clinical samples using multiplexed fluorescence immunohistochemistry (mfIHC) and digital image analysis. Effect on survival was assessed using Cox regression analyses. The outcome measurement was all‐cause mortality. Univariate analysis revealed that high expression of secreted protein acidic and cysteine rich (SPARC) and fibroblast activation protein in stromal cells was associated with shorter survival. Importantly, high expression of platelet‐derived growth factor receptor beta (PDGFRB) in tumor cells, but not in stromal cells, was associated with shorter survival (hazard ratio [HR] = 1.02, *p* < 0.001). A multivariable survival analysis adjusted for clinical parameters and stromal mfIHC markers revealed that tumor cell PDGFRB and stromal SPARC remained independently associated with survival (HR = 1.01, 95% confidence interval [CI] = 1.00–1.03 and HR = 1.05, 95% CI = 1.00–1.11, respectively). The prognostic effect of PDGFRB was validated with an artificial intelligence‐based analysis method and further externally validated in another cohort of 117 MPM patients. In external validation, high tumor cell PDGFRB expression associated with shorter survival, especially in the epithelioid subtype. Our findings suggest PDGFRB and SPARC as potential markers for risk stratification and as targets for therapy.

## Introduction

Malignant pleural mesothelioma (MPM) is a tumor originating from the mesothelial cells lining the pleural cavity. MPM has a poor prognosis; the median MPM patient survival time is 10 months [1]. The main cause of MPM is exposure to asbestos and curative treatment is usually limited. Factors associated with long‐term survival (>60 months) in MPM patients remain unidentified [2].

The amount of stromal fibroblasts in malignant tissues and their molecular composition may have a significant prognostic role in multiple types of malignancies [3, 4, 5, 6, 7]. Furthermore, cancer‐associated fibroblasts (CAFs) have been identified as potential targets in preclinical *in vitro* and *in vivo* studies, although the clinical efficacy of targeting CAFs has not yet been reported [4, 8, 9]. In MPM, the composition of stromal fibroblasts, their significance in patient survival, and their potential as possible therapeutic targets have not been systematically investigated, although recent studies have shown that the histological features associated with poor prognosis are localized particularly in the MPM tumor stroma [10]. However, most of the studies that investigated stromal fibroblasts in MPM were conducted using *in vitro* models and the spatial characteristics of MPM tumor cells and their surrounding fibroblasts remain poorly characterized [11, 12].

Multiplexed fluorescence immunohistochemistry (mfIHC) is a histopathological technique that enables the detection of multiple protein markers and nuclei simultaneously [13]. Compared to conventional single‐marker immunohistochemistry (IHC), mfIHC enables automated tissue component or cell class‐specific expression analysis, thus allowing a better spatial understanding of complex pathological processes. For example, mfIHC has been used in prostate cancer to identify subtypes of stromal fibroblasts strongly prognostic of patient survival [3]. However, mfIHC has not been used to study fibroblast markers in MPM.

MPM tumor cells are known to undergo a mesenchymal transition with gene expression and morphological alterations associated with poor prognosis [14, 15, 16]. We hypothesized that, with mfIHC, we could identify and quantify mesenchymal markers in both MPM tumor cells and their surrounding stromal cells and correlate their expression with patient survival. To test this hypothesis, we profiled the expression and distribution of a set of known mesenchymal markers both in tumor cells and in stromal fibroblasts and then validated the most robust prognostic phenotype using an artificial intelligence (AI) model and a validation cohort.

## Materials and methods

### Patients

The study sample consisted of 74 patients from a Finnish national MPM population diagnosed during 2000–2012 [1]. From the national MPM tissue sample cohort, subgroups of long‐term MPM survivors (LTS, survival >60 months) [2, 17], epithelioid MPM patients (EMPM, median survival 14 months) [2, 17], extended pleurectomy MPM patients (PD), and biphasic MPM patients (BMPM) with histological samples in the Helsinki Biobank were included for constructing tissue microarrays (TMAs). The TMAs were constructed in collaboration with the Helsinki Biobank. The ethics committee of Helsinki University Hospital approved the study (HUS/1057/2019).

### Tissue samples, histopathological evaluation, TMAs, and clinical data

Detailed diagnostic information for the LTS and EMPM groups has been described previously [2, 17]. In the PD group, samples were obtained from extended pleurectomy procedures and diagnoses were verified by HW. The diagnoses were also verified by HW in the BMPM group. Samples with sufficient tumor tissue were selected for TMAs, following scanning of hematoxylin and eosin‐stained slides and digital annotation. Annotations included tumor foci (2 cores per patient) and benign foci outside the tumor (1–2 cores per patient). In the BMPM group, the tumor foci were annotated separately from epithelial and sarcomatoid areas (2 cores per area). The benign foci included fat, muscle, or lung tissue. The diameter of one TMA core was 1.0 mm.

Clinical data were collected from the Helsinki University Hospital medical records. The clinical stage was defined based on the eighth edition of the TNM (tumor, lymph nodes, metastasis) classification for MPM [18]. Asbestos exposure status was obtained through the occupational disease register and medical records, based on visits with pulmonologists or occupational disease experts. Smoking status was obtained through medical records.

### mfIHC and panels

Fibroblast markers and their distribution in tumor stroma and the relationship between fibroblasts and tumor cells were assessed using two mfIHC panels, including the following seven fibroblast markers: platelet‐derived growth factor receptor alpha (PDGFRA), platelet‐derived growth factor receptor beta (PDGFRB), alpha smooth muscle actin (aSMA) and fibroblast activation protein (FAP) in panel 1, and secreted protein acidic and cysteine rich (SPARC, also known as osteonectin or basement membrane protein 40), periostin (POSTN), and collagen I in panel 2 [4, 19]. Joensuu *et al* have shown that the mRNAs of SPARC, collagen I, and POSTN are highly expressed in human MPM tissues (personal communication); therefore, we included these markers in panel 2. For panel 1, cytokeratin 5 (CK5) and for panel 2, a combination of CK5, CK5/6, and calretinin antibodies were used to detect MPM tumor cells. Detailed information regarding the staining procedure, antibodies, and imaging is presented in supplementary material, File S1 [15, 20].

### Digital pixel‐based image analysis

Detailed information regarding digital pixel‐based image analysis and quality control of the TMA spots is presented in supplementary material, File S2 [21, 22, 23]. In brief, the scanned and exported images were cropped to individual TMA spots and the spots from the first and second staining rounds were overlaid. Next, we used a machine‐learning based approach to mask autofluorescence, blood vessels, background, and tissue (all the remaining signal excluding the features described before) from the images (Ilastik [version 1.3.3post1 for MacOS]) [22]. Furthermore, we used the tissue and the blood vessel masks in the final image analysis pipeline (CellProfiler [version 3.1.9]) [23]. In the final pipeline, the masked tissue was further classified into different tissue components (mesothelioma [tumor cells], total stroma, and different stromal components; meso zones 1–4 and vessel zones 1–4) and the mean intensity (also referred to as ‘expression’ in the text) of each single channel was measured in these components.

**Figure 1:**
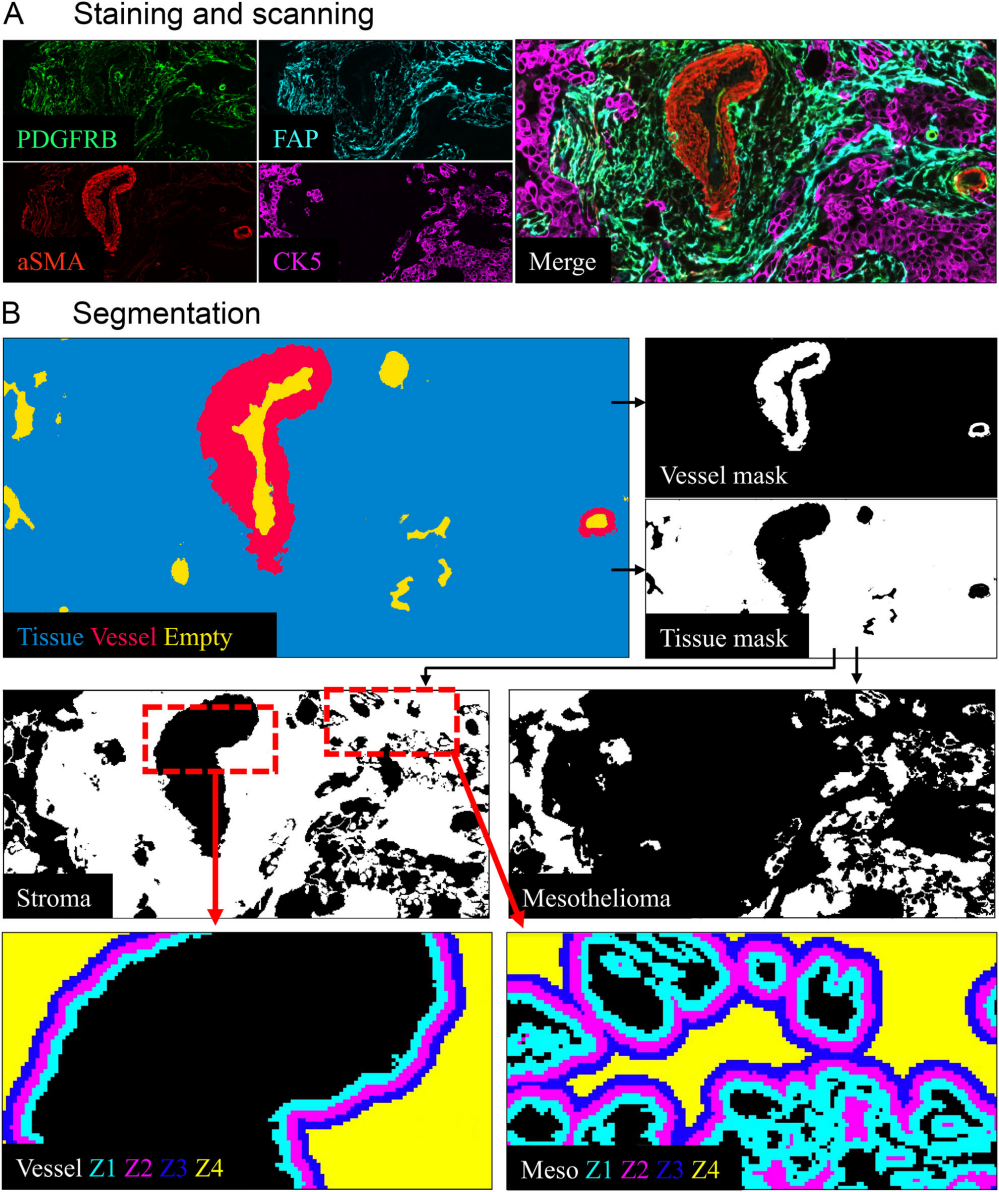
Study workflow. (A) mfIHC staining and scanning. (B) Image segmentation to tissue (blue, excluding vessels), vessels (red), and empty (yellow). Tissue was segmented to the stromal component (shown in white on the left‐hand side) and to the mesothelioma component (shown in white on the right‐hand side). Stroma was further segmented into stromal zones around vessels (Vessel Z1–Z4) and stromal zones around mesothelial cells (Meso Z1–Z4).

The workflow is shown in Figure 1.

### Statistical analyses

For the statistical analyses, R (R Core Team [2017], Vienna, Austria) and SPSS Statistics (version 25.0; IBM, Armonk, NY, USA) were used. Categorical variables are presented as numbers with percentages. Continuous data were evaluated for skewness by using histograms. Because all continuous variables were skewed, data are reported as medians with interquartile ranges (IQR) and compared between groups using a nonparametric Mann–Whitney *U*‐test. The Spearman's rank correlation coefficient was used to assess correlation between continuous nonparametric variables. The log‐rank test was used to compare survival times between groups.

Univariate Cox regression analysis was used to identify fibroblast markers associated with patient survival. Prior to Cox regression, the average mean intensity variables were multiplied by 1,000 to make the hazard ratios (HR) more relevant [24]. Bonferroni correction was performed to adjust for multiple comparisons.

Multivariable Cox regression analysis was performed to study survival in relation to other known prognostic factors. The multivariable Cox regression analysis was adjusted for variables associated with survival (*p* < 0.05) and previously known predictors: age, sex, side of the disease, histology, and TNM stage [1, 25]. Again, the average mean intensity variables were multiplied by 1,000 to make the HRs more relevant [24]. The proportional hazards assumption was tested by assessing the relationship between Schoenfeld residuals and time.

Survival time was calculated as the time from pathological diagnosis (the date the diagnostic tissue sample was taken) to the date of death. Three patients were still alive at the end of follow‐up (2 July 2019).

### *Post hoc* validation of the prognostic value of tumor cell PDGFRB in MPM using conventional IHC and AI analysis

As mfIHC is not commonly available in diagnostic laboratories, we tested whether AI‐based PDGFRB analysis (which may be implemented into clinical practice) would also be of prognostic relevance. Therefore, we analyzed the relationship between the area (in comparison to intensity) of PDGFRB‐positive MPM tumor cells and survival using conventional IHC and AI analysis.

**Figure 2:**
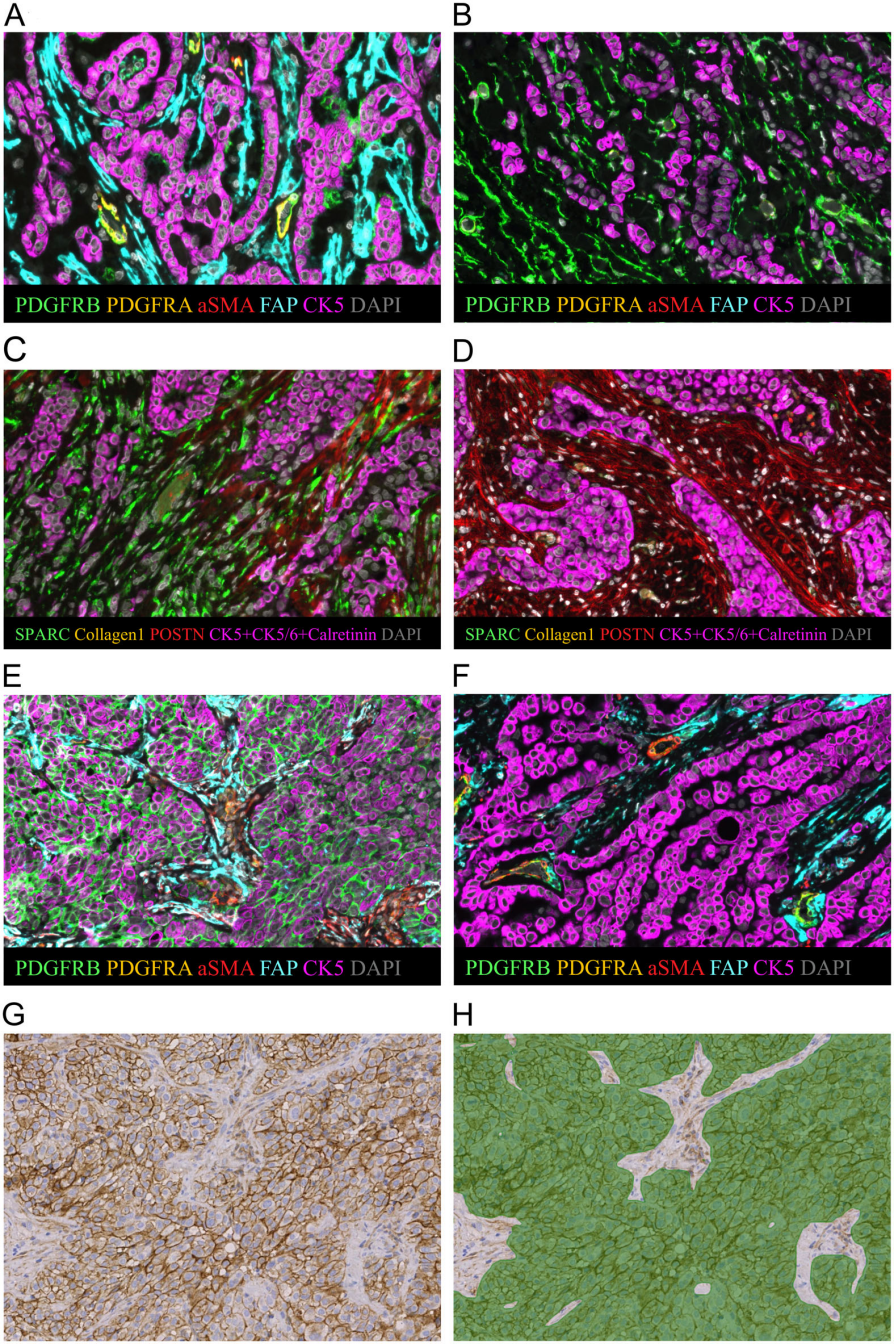
Example images. Sample with (A) high stromal FAP intensity, (B) low stromal FAP intensity, (C) high stromal SPARC intensity, (D) low stromal SPARC intensity, (E) high tumor cell PDGFRB intensity, (F) low tumor cell PDGFRB intensity, (G) PDGFRB DAB staining, and (H) PDGFRB‐positive tumor cells (in green) identified by the trained AI model (Aiforia® platform).

PDGFRB DAB (3,3′‐diaminobenzidine) staining of the TMA slides was first performed. Following digital scanning of the slides, we trained an AI model using a deep convolutional neural network method (Aiforia® Technologies, Helsinki, Finland) to detect the PDGFRB‐positive MPM tumor cells. We trained the AI model to detect good quality tissue (excluding folded tissue), PDGFRB‐negative MPM tumor cells, and PDGFRB‐positive MPM tumor cells (Figure 2). A tumor cell was classified as PDGFRB‐positive if any PDGFRB cell membrane staining was registered.

The areas of tissue, PDGFRB‐positive tumor cells, and PDGFRB‐negative tumor cells were measured. The areas that the AI model detected with a class confidence ≥85% were included in the final analysis. Next, the area of the PDGFRB‐positive tumor cells was set in proportion to the total tumor cell area (including PDGFRB‐positive and ‐negative tumor cells). The same statistical analyses were applied to the *post hoc* analysis as in the primary analysis. Furthermore, the AI model results were compared to the mfIHC results by calculating Spearman's rank correlation coefficient.

### *Post hoc* association analyses in the primary cohort

We performed *post hoc* analyses investigating the association between high tumor cell PDGFRB expression and tumor size, mitoses, nuclear grading, and tumor architecture, as these factors have been previously shown to be associated with epithelioid MPM prognosis [26, 27, 28]. These factors were available for those 66 patients with epithelioid mesotheliomas who were included from our previous study [2, 17]. We also analyzed the association of BAP1 tumor status and tumor cell PDGFRB expression. The variable definitions can be found in supplementary material, File S3.

We tested for association between continuous nonparametric variables using the Spearman's rank correlation test and between categorical variables using a two‐sided *χ* ^2^ test. We also performed univariate Cox regression analyses to assess whether these variables were associated with survival. Finally, we added the variables that were significantly associated with survival in univariate analysis into multivariable Cox regression model to identify variables independently associated with survival.

### *Post hoc* validation of PDGFRB in an external validation cohort

To externally validate the negative prognostic value of tumor cell PDGFRB, we used a validation TMA cohort of 117 Finnish MPM patients. The cohort had been compiled by SA, EK, and HW at the Finnish Institute of Occupational Health [29]. The samples were collected from Central Hospitals in Finland, the patients were diagnosed between 1990 and 2006, and the diagnoses were verified by expert pathologists. The cohort included 76 (65%) epithelioid, 19 (16%) biphasic, and 22 (19%) sarcomatoid mesotheliomas. The median time to death was 10 months, the median age at the time of diagnosis was 63 years, and 11% of the patients were female. Please see Table 1 in supplementary material, File S4 for detailed information regarding patient characteristics.

Fresh tissue sections from the TMA blocks were cut, stained with PDGFRB DAB, and scanned. For detecting the PDGFRB‐positive tumor cells, the same AI model as used for the primary cohort was further trained to detect the good quality tissue (excluding folded tissue), PDGFRB‐negative tumor cells, and PDGFRB‐positive tumor cells from the scanned TMA images (see Figure 1 in supplementary material, File S4). The analyses were applied similarly as for the primary study cohort (see above).

Permission to use the TMA blocks in the validation cohort was obtained from Valvira National Supervisory Authority for Welfare and Health DnroV/44410/2019 (Dnro 5929/06.01.03.01/2013).

## Results

### Patient characteristics

Patient characteristics are described in Table 1. The primary study material consisted of 74 MPM patients, of whom 16 (22%) survived longer than 60 months. All these long‐term survivors had the epithelioid type of MPM. None of the investigated factors showed an association with overall survival (Table 1).

**Table 1:** Patient characteristics and median time to death according to baseline characteristics. Primary cohort.

Variable	Prevalence (*n* = 74)	Months to death from diagnosis* (IQR)	*P* value^†^
**All patients**	NA	18.0 (8.3–41.7)	NA
**Age** (years) at the time of diagnosis, median (IQR)	65 (59–72)		
≤65	36 (49%)	16.6 (8.2–46.1)	0.233
>65	38 (51%)	19.5 (8.3–38.6)	
**Sex**			
Female	12 (16%)	24.5 (18.0–58.8)	0.379
Male	62 (84%)	16.3 (8.0–35.2)	
**Histology**			
Epithelioid	69 (93%)	17.8 (8.4–42.9)	0.355
Mixed (biphasic)	5 (7%)	23.4 (3.8–37.3)	
**Exposure to asbestos**			
Yes	52 (70%)	16.6 (7.1–29.8)	0.149
No	22 (30%)	22.0 (11.1–60.0)	
**Smoking**			
Current smoker	8 (11%)	35.3 (4.9–80.8)	0.801
Ex‐smoker	29 (39%)	20.7 (10.9–49.5)	
Never smoker	37 (50%)	16.6 (6.9–24.5)	
**Smoking pack years**^‡^, **median (IQR)**	30 (15–40)		
1–30	20 (27%)	24.3 (11.1–65.3)	0.647
>30	15 (20%)	15.7 (9.5–56.0)	
**Treatment** ^§^			
Only chemotherapy	20 (27%)	16.3 (10.9–35.2)	NA
Only surgery	14 (19%)	9.1 (4.3–31.0)	
Chemotherapy and surgery	24 (32%)	24.1 (19.4–51.1)	
Radiation therapy	7 (9%)	50.5 (13.7–78.9)	
**Stage** ^¶^			
I	27 (37%)	24.2 (8.0–57.1)	0.405
II	5 (7%)	29.8 (2.7–82.3)	
III	29 (40%)	12.3 (8.3–24.4)	
IV	12 (16%)	16.6 (11.8–24.1)	

^*^ Three patients were still alive at the end of follow‐up.

^**†**^ Tested using a log‐rank test.

^**‡**^ For smokers or ex‐smokers, data were missing for two patients.

^§^ One patient can belong to several groups. No *P* value test.

^¶^ Data were missing for one patient.

### Individual fibroblast markers and survival

We first measured the expression (intensity) of the seven fibroblast markers separately in tumor cell and stromal cell components (see supplementary material, File S5A). We hypothesized that the expression of fibroblast markers within stroma could depend on the distance from the mesothelial tumor cells or vessels. However, there were no notable differences in the distribution of stromal marker expression away from the mesothelium (meso zones 1–4; zone = 12 μm) or away from the vessels (vessel zones 1–4) (see supplementary material, File S5B,C). There were no differences in fibroblast marker expression between the epithelioid and sarcomatoid components in biphasic tumors (see Figures A and B, and Table in supplementary material, File S6).

Of the investigated fibroblast markers, high expression of FAP and SPARC in tumor stroma and high expression of PDGFRB in MPM tumor cells were associated with shorter survival (Table 2). However, after correcting for multiple comparisons, only PDGFRB displayed a statistically significant association with survival. In contrast, PDGFRA, aSMA, collagen‐1, and POSTN did not display any association with survival in univariate analysis (see Table in supplementary material, File S7).

**Table 2:** Univariate Cox regression analysis. Primary cohort.

mfIHC variable	*P* value	HR	*P* corr
FAP_stroma	0.03*	1.01	1.00
FAP_meso_Z1	0.04*	1.01	1.00
FAP_meso_Z2	0.03*	1.01	0.91
FAP_meso_Z3	0.04*	1.01	1.00
FAP_meso_Z4	0.02*	1.01	1.00
FAP_vessel_Z1	0.05	1.00	1.00
FAP_vessel_Z2	0.04*	1.00	1.00
FAP_vessel_Z3	0.02*	1.01	1.00
FAP_vessel_Z4	0.05	1.01	1.00
PDGFRB_meso	0.0006***	1.02	0.04*
SPARC_stroma	0.006**	1.07	0.21
SPARC_meso_Z1	0.005**	1.06	0.16
SPARC_meso_Z2	0.002**	1.07	0.08
SPARC_meso_Z3	0.003**	1.07	0.11
SPARC_meso_Z4	0.007**	1.07	0.26

An HR > 1 indicates an increased risk of death and HR < 1 indicates a decreased risk of death. mfIHC variables are average mean intensities in mesothelial and stromal tissue components. Average mean intensities were multiplied by 1,000. Only variables with significant *P* value are shown. Other variables are found in supplementary material, File S7.

meso_Z1–Z4, meso zones 1–4; *P* corr, Bonferroni‐corrected *P* value; vessel_Z1–Z4, vessel zones Z1–Z4.

* *p* < 0.05; **p < 0.01; ***p < 0.001.

### FAP and SPARC expression in tumor stroma

In univariate Cox regression using continuous values, higher FAP expression (HR = 1.01, *p* = 0.03, corrected *P* value = 1.00) and higher SPARC expression (HR = 1.07, *p* = 0.006, corrected *P* value = 0.21) in tumor stroma were associated with shorter survival in MPM patients (Figure 2A–D).

Interestingly, stromal SPARC expression correlated negatively with the MPM tumor cell marker staining (combination of CK5, calretinin, and CK5/6) (Spearman's rho: −0.432; *p* = 0.001) and positively with tumor cell PDGFRB expression (Spearman's rho: 0.314; *p* = 0.008). There was a statistically significant correlation between stromal FAP and stromal SPARC (Spearman's rho: 0.380; *p* = 0.001).

### PDGFRB expression in tumor cells

In univariate Cox regression, higher PDGFRB expression in MPM tumor cells was associated with shorter survival in MPM patients (HR = 1.02, *p* < 0.001, corrected *P* value = 0.04) (Figure 2E,F).

### Multivariable analysis

We used multivariable Cox regression analysis separately for the fibroblast markers while adjusting for age, sex, side of the disease, clinical stage, and histology. High tumor cell PDGFRB and high stromal SPARC were independently associated with survival (Table 3). After combining all three markers into one multivariable model, high tumor cell PDGFRB (HR = 1.01, 95% confidence interval [CI] = 1.00–1.03; *p* = 0.005) and high stromal SPARC (HR = 1.05, 95% CI = 1.00–1.11; *p* = 0.045) remained independently associated with survival. Subgroup analysis including only those with epithelioid histology found high PDGFRB to associate with an increased risk of death (HR = 1.01, 95% CI = 1.00–1.03, *p* = 0.006; Table 3).

**Table 3:** Multivariable Cox regression analysis. Primary cohort.

Variable	HR (95% CI)	*P* value
**Individual markers (*n* = 69**^†^)		
Tumor cell PDGFRB mean intensity	1.02 (1.01–1.03)	0.001***
Stromal FAP mean intensity	1.00 (1.00–1.01)	0.113
Stromal SPARC mean intensity	1.09 (1.03–1.14)	0.001***
**All markers combined (*n* = 69**^†^)		
Age	1.02 (0.99–1.05)	0.135
Sex		
Male	1.0	
Female	0.71 (0.35–1.46)	0.351
Side of the disease		
Right	1.0	
Left	0.61 (0.36–1.04)	0.071
Clinical stage		
Low	1.0	
High	1.47 (0.87–2.54)	0.148
Histology		
Epithelioid	1.0	
Biphasic	1.17 (0.43–3.16)	0.759
Tumor cell PDGFRB mean intensity	1.01 (1.00–1.03)	0.005**
Stromal FAP mean intensity	1.00 (1.00–1.01)	0.299
Stromal SPARC mean intensity	1.05 (1.00–1.11)	0.045*
**Only epithelioid cases (*n* = 64**^†^)		
Age	1.01 (1.00–1.04)	0.283
Sex		
Male	1.0	
Female	0.65 (0.31–1.35)	0.247
Side of the disease		
Right	1.0	
Left	0.69 (0.40–1.19)	0.180
Clinical stage		
Low	1.0	
High	1.73 (0.95–3.15)	0.074
Tumor cell PDGFRB mean intensity	1.01 (1.00–1.03)	0.006**
Stromal FAP mean intensity	1.00 (1.00–1.01)	0.260
Stromal SPARC mean intensity	1.05 (1.00–1.11)	0.084

Multivariable Cox regression adjusted for age, sex, side of the disease, clinical stage, and histology. An HR > 1 indicates an increased risk of death and an HR < 1 indicates a decreased risk of death. All models fulfilled the proportional hazard assumption.

* *p* < 0.05; **p < 0.01; ***p < 0.001.

^†^ TNM staging was missing for one patient.

### *Post hoc* validation of the prognostic value of tumor cell PDGFRB in MPM using conventional IHC and AI analysis

To validate tumor cell PDGFRB as a prognostic factor, we used an AI‐based deep convolutional neural network method (see *Materials and methods*). The trained AI model detected PDGFRB‐positive tumor cells in 33 out of 74 patients (45%). The ratio between PDGFRB‐positive tumor cell area and total tumor cell area ranged from 0.1 to 98.1%, with a median of 1.9% (IQR: 0.3–13.5%). Univariate Cox regression showed that higher tumor cell PDGFRB associated with shorter survival with an HR of 4.48 (95% CI = 1.34–14.99; *p* = 0.015) (Figure 2G,H). Multivariable Cox regression adjusted for age, sex, side of the disease, clinical stage, and histology revealed that PDGFRB was associated with an increased risk of death (HR = 6.19, 95% CI = 1.77–21.63; *p* = 0.004). The Spearman's rank correlation coefficient between the mfIHC (tumor cell PDGFRB intensity) and AI results (tumor cell PDGFRB area) was 0.489 (*p* < 0.001).

### *Post hoc* association analyses in the primary cohort

No statistically significant associations were observed between tumor size, clinical stage, nuclear grading, mitoses or BAP1 status, and tumor cell PDGFRB expression (see Table 1 in supplementary material, File S3). However, there was a positive association between unfavorable tumor architecture (predominantly solid or micropapillary growth pattern) and high tumor cell PDGFRB expression (*p* = 0.020) (see Table 1 and Figures 1 and 2 in supplementary material, File S3). In univariate Cox regression analysis, high nuclear grading (HR = 1.95, *p* = 0.001), unfavorable tumor architecture (HR = 2.69, *p* = 0.001), high average mitotic count (HR = 1.12, *p* = 0.003), and BAP1 tumor positivity (HR = 1.80, *p* = 0.030) were associated with shorter survival (see Table 1 in supplementary material, File S3). In multivariable analysis, unfavorable tumor architecture (HR = 3.20, 95% CI = 1.32–7.76; *p* = 0.010) and high tumor cell PDGFRB expression (HR = 1.02, 95% CI = 1.00–1.03; *p* = 0.006) were independently associated with shorter survival (see Table 2 in supplementary material, File S3). We also looked into the prognostic value of tumor cell PDGFRB separately in tumor architecture groups. Even though tumor cell PDGFRB expression was higher in the unfavorable tumor architecture group, the negative effect of high tumor cell PDGFRB on survival was seen in both groups (see Table 3 in supplementary material, File S3).

### *Post hoc* validation of PDGFRB in an external validation cohort

In univariate Cox regression analysis, high relative tumor PDGFRB area was associated with shorter survival (HR = 2.10, *p* = 0.010, corrected *P* value = 0.041) in an independent validation cohort (see Table 2 in supplementary material, File S4). In multivariable Cox regression analysis, adjusted for age, sex, and histology, no association between tumor PDGFRB area and survival was noted (HR = 1.25, 95% CI = 0.66–2.35; *p* = 0.489) (see Table 3 in supplementary material, File S4). After including only epithelioid subtypes, no clear association was seen, although the point estimate direction was the same as in the primary cohort (HR = 2.13, 95% CI = 0.84–5.40; *p* = 0.112; see Table 4 in supplementary material, File S4). There was no clear difference in relative tumor PDGFRB area and histology due to the large variation and limited sample size (see Figure 2 in supplementary material, File S4).

## Discussion

### Key findings

In this study, we have shown that high FAP and SPARC expression in tumor stroma and high PDGFRB expression in tumor cells are associated with shorter survival in MPM patients in univariate analysis. We observed a statistically significant correlation between tumor cell PDGFRB and stromal SPARC expression, whereas high stromal SPARC expression correlated inversely with the expression of mesothelial marker staining (combination of CK5, calretinin, and CK5/6). Furthermore, we observed a correlation between stromal SPARC and stromal FAP. Thus, after adjusting for known prognostic factors and including all three markers in multivariable analysis, only tumor cell PDGFRB and stromal SPARC had independent statistically significant associations with survival.

Due to the statistically stronger correlation of tumor cell PDGFRB and survival, we validated its prognostic value using an AI model (Aiforia® platform) and showed that a high relative PDGFRB tumor cell positivity correlated with shorter survival. Finally, we performed an external validation with an independent validation cohort. In univariate analysis, we showed that there was an association between high tumor cell PDGFRB expression and shorter survival. However, in the multivariable analysis, high relative tumor PDGFRB area was not independently associated with overall survival. We hypothesized that this might be a consequence of the sarcomatoid cases included in the validation cohort. However, no significant association was found after excluding the sarcomatoid and the biphasic subtypes (i.e. only including the epithelioid subtype) from the analysis. Thus, the negative prognostic value of PDGFRB in MPM appears more conclusive in the primary cohort than in the validation cohort; however, this may be a result of inadequate statistical power in the validation cohort. In addition, the validation cohort included only one patient with a survival longer than 60 months.

### Comparison to previous literature

PDGFRB is a receptor tyrosine kinase (RTK) known to be expressed by stromal fibroblasts in several cancers and high stromal PDGFRB expression is associated with poor survival, e.g. in breast and prostate cancers [30, 31, 32, 33, 34]. Consistent with the current study, in MPM, PDGFRB is expressed in both stromal fibroblasts and MPM tumor cells [35]. Approximately 30–50% of MPMs express PDGFRB [36, 37, 38] (45% in this study) and the expression is dominant in epithelial and biphasic subtypes of MPM [36, 38].

By using two different PDGFRB staining and analytical methods, we conclude that high tumor cell PDGFRB expression, measured either as intensity (fluorescence staining) or relative positive area (DAB staining), is associated with shortened survival in MPM patients. Our results are consistent with an earlier study, where it was shown in a cohort of 48 patients that PDGF receptor signaling pathways were differentially activated in MPM patients who survived less than 3 years compared with those who survived more than 3 years [39]. Furthermore, Tsao *et al* showed in a cohort of 24 MPM patients that high baseline PDGFRB expression in cytoplasm, stroma, and nucleus correlated with shorter progression‐free survival but was not statistically significantly associated with overall survival [40]. In another study by the same group, PDGFRB IHC expression was not found to be prognostic of survival in 17 MPM patients [41]. In summary, previous studies on PDGFRB expression and MPM survival are not conclusive. Our study is the first to show the effect of tumor cell‐specific PDGFRB expression on patient overall survival in two larger patient cohorts. We used two different unbiased computerized scoring methods and showed independent prognostic value in the primary cohort, but not in the validation cohort. In our external validation analysis, including only epithelioid tumors, the point estimate direction was the same as in the primary cohort, although it was not statistically significant.

PDGFRB expression in MPM tumor cells provides a potential target for tyrosine kinase inhibitors (TKIs). However, the more unselective TKIs such as dasatinib and axitinib have not shown any effect on MPM patient survival in phase I–II trials [40, 42]. Furthermore, the more selective TKI imatinib mesylate (IM, also known as Gleevec® or Glivec®, Novartis Pharmaceuticals Corporation, Basel, Switzerland) has similarly showed weak results [41, 43] even when targeting the treatment to a specific subgroup of patients expressing PDGFRB at baseline [37, 44, 45]. It is possible that IM (targeting the Abelson proto‐oncogene, C‐kit, and PDGFRs) is an insufficiently selective PDGFRB inhibitor and novel, more selective, inhibitors should be investigated in clinical trials. It may also be that multiple RTKs (including PDGFRB) should be targeted simultaneously in the treatment of MPM instead of targeting a single RTK [46]. Still, given the strong association between PDGFRB and survival in our study, clinical trials including more patients with more specific PDGFRB inhibitors and AI‐based PDGFRB *in situ* analysis of patient tumor tissue are warranted.

In the primary cohort analysis, stromal SPARC also associated with shorter survival. SPARC is a calcium‐binding matricellular protein typically expressed in mineralized tissues [47]. Little is known about the role of SPARC in MPM. SPARC is expressed in MPM cell lines and patient tissue and blood samples [48, 49]. In tissue samples, SPARC expression has been detected in stromal fibroblasts and MPM tumor cells [49]. We observed SPARC expression in both. Interestingly, we also observed that tumors expressing high stromal SPARC expressed faint or no mesothelial marker staining (combination of CK5, calretinin, and CK5/6). However, whether this indicates that MPM tumor tissue is transforming into a sarcomatoid or mesenchymal direction due to high stromal SPARC expression or if high stromal SPARC is already present in more sarcomatoid‐like MPM tumors remains speculative. Furthermore, we found an association between high stromal SPARC expression and shorter survival in univariate and multivariable analyses in the primary cohort. This finding adds strength to the prognostic value of SPARC in MPM and highlights its attractiveness for further investigation.

### Strengths and limitations

This study has some strengths. Instead of conventional IHC, we used mfIHC to analyze the fibroblast markers, which enabled investigation of multiple different markers simultaneously and their distribution in different tissue components. This is a novel approach that has not previously been widely used for investigating the MPM tumor microenvironment. We performed a *post hoc* validation of the PDGFRB finding using an AI model based on a convolutional neural network method. In comparison to previous studies, our study included a rather large sample size with previously confirmed MPM cases. As the statutory population registry in Finland captures all deaths, we had full follow‐up for the entire cohort. Finally, we validated our results in an external validation cohort of 117 previously confirmed MPM patients.

There were some limitations that should be acknowledged. Our cohort is from a rather genetically homogeneous single‐nation population, which may limit the generalizability to other countries. Some patients were diagnosed nearly 20 years ago, and both diagnostic and treatment MPM strategies have developed since. Our study population represented a selected subpopulation of MPM patients who were fit enough to undergo biopsy or surgery, as patients diagnosed with MPM at autopsy were not included in the study. Thus, median survival in our study was higher than in population‐based epidemiological studies [1, 25]. The fact that tumor PDGFRB independently predicted overall survival in the primary cohort, but not in the validation cohort, could be related to the differences in the patient populations in these cohorts or due to lack of power. The primary cohort included a selected subpopulation of long‐term survivors. The vast majority of our patients, especially in the primary cohort, had the epithelial subtype of MPM, and our results may not be generalizable to other MPM subtypes. Elevated blood platelet count is associated with poor overall survival in MPM [50]. It is possible that a high blood platelet count indicates higher tumor cell PDGFRB expression. The correlation between platelet count and tumor cell PDGFRB warrants further studies.

## Conclusions

In conclusion, in the primary cohort of MPM patients including long‐term survivors, high FAP and SPARC expression in stromal cells and high PDGFRB expression in tumor cells were associated with shorter survival. Tumor cell PDGFRB and stromal SPARC expression in particular were associated with shorter survival and may play critical roles in the pathogenesis of MPM. This positive expression correlation suggests that they may also be regulated by common signals.

After validation, we conclude that tumor PDGFRB expression was seen in all histological subtypes. In the epithelioid subtype, PDGFRB expression was associated with solid or micropapillary tumor architecture. High PDGFRB expression in mesothelioma tumor cells is a negative prognostic marker, especially in patients with epithelioid histology. Thus, PDGFRB is a potential marker for risk stratification and a target for therapy in MPM. Further studies focusing on patients with high PDGFRB expression are warranted.

## Author contributions statement

HO analyzed the data, performed the statistical analyses, and wrote the paper. JP collected the clinical data regarding the primary study cohort. HW and ES gathered the primary study cohort and were responsible for constructing the TMAs. HW, SA and EK gathered the validation cohort and constructed the TMAs. KV coordinated and carried out the mfIHC experiments. AO together with TP designed the mfIHC panels. OK, MM and JR provided their special expertise for designing and conducting the study. II, MIM and TP were responsible for the study design and supervised the project. All authors provided critical feedback and contributed to the writing of the final manuscript.

## Supporting information

**File S1.** Description of staining procedure, antibodies, and imaging

**File S2.** Detailed information about digital pixel‐based image analysis and quality control of the TMA spots

**File S3.** Primary cohort *post hoc* association analyses

**File S4.** External validation

**File S5.** Marker mean intensity distributions in mesothelial and stromal components, meso zones 1–4 and vessel zones 1–4

**File S6.** Biphasic mesotheliomas in the primary cohort

**File S7.** Univariate Cox regression analysis showing the association between individual markers and survival

## Data Availability

The image analysis pipeline files are available upon request.

## References

[1] Laaksonen S, Ilonen I, Kuosma E, *et al*. Malignant pleural mesothelioma in Finland: regional and gender variation. Acta Oncol 2019; 58: 38–44. 30375909 10.1080/0284186X.2018.1532599

[2] Paajanen J, Laaksonen S, Kettunen E, *et al*. Histopathological features of epithelioid malignant pleural mesotheliomas in patients with extended survival. Hum Pathol 2020; 98: 110–119. 32142836 10.1016/j.humpath.2020.02.007

[3] Blom S, Erickson A, Östman A, *et al*. Fibroblast as a critical stromal cell type determining prognosis in prostate cancer. Prostate 2019; 79: 1505–1513. 31269283 10.1002/pros.23867 PMC6813917

[4] Öhlund D, Elyada E, Tuveson D. Fibroblast heterogeneity in the cancer wound. J Exp Med 2014; 211: 1503–1523. 25071162 10.1084/jem.20140692 PMC4113948

[5] Kawase A, Ishii G, Nagai K, *et al*. Podoplanin expression by cancer associated fibroblasts predicts poor prognosis of lung adenocarcinoma. Int J Cancer 2008; 123: 1053–1059. 18546264 10.1002/ijc.23611

[6] Ren J, Smid M, Iaria J, *et al*. Cancer‐associated fibroblast‐derived Gremlin 1 promotes breast cancer progression. Breast Cancer Res 2019; 21: 109. 31533776 10.1186/s13058-019-1194-0 PMC6751614

[7] Gerstenberger W, Wrage M, Kettunen E, *et al*. Stromal caveolin‐1 and caveolin‐2 expression in primary tumors and lymph node metastases. Anal Cell Pathol 2018; 2018: 8651790. 10.1155/2018/8651790 PMC5914130 29850392

[8] Pietras K, Östman A. Hallmarks of cancer: interactions with the tumor stroma. Exp Cell Res 2010; 316: 1324–1331. 20211171 10.1016/j.yexcr.2010.02.045

[9] Valkenburg KC, de Groot AE, Pienta KJ. Targeting the tumour stroma to improve cancer therapy. Nat Rev Clin Oncol 2018; 15: 366–381. 29651130 10.1038/s41571-018-0007-1 PMC5960434

[10] Courtiol P, Maussion C, Moarii M, *et al*. Deep learning‐based classification of mesothelioma improves prediction of patient outcome. Nat Med 2019; 25: 1519–1525. 31591589 10.1038/s41591-019-0583-3

[11] Kanaji N, Kita N, Kadowaki N, *et al*. Fibronectin and hepatocyte growth factor produced by lung fibroblasts augment migration and invasion of malignant pleural mesothelioma cells. Anticancer Res 2017; 37: 2393–2400. 28476806 10.21873/anticanres.11578

[12] Li Q, Wang W, Yamada T, *et al*. Tumorigenesis and neoplastic progression pleural mesothelioma instigates tumor‐associated fibroblasts to promote progression via a malignant cytokine network. Am J Pathol 2011; 179: 1483–1493. 21763682 10.1016/j.ajpath.2011.05.060 PMC3157262

[13] Blom S, Paavolainen L, Bychkov D, *et al*. Systems pathology by multiplexed immunohistochemistry and whole‐slide digital image analysis. Sci Rep 2017; 7: 15580. 29138507 10.1038/s41598-017-15798-4 PMC5686230

[14] Schramm A, Opitz I, Thies S, *et al*. Prognostic significance of epithelial‐mesenchymal transition in malignant pleural mesothelioma. Eur J Cardiothorac Surg 2010; 37: 566–572. 19781955 10.1016/j.ejcts.2009.08.027

[15] Fassina A, Cappellesso R, Guzzardo V, *et al*. Epithelial‐mesenchymal transition in malignant mesothelioma. Mod Pathol 2012; 25: 86–99. 21983934 10.1038/modpathol.2011.144

[16] de Reyniès A, Jaurand MC, Renier A, *et al*. Molecular classification of malignant pleural mesothelioma: identification of a poor prognosis subgroup linked to the epithelial‐to‐mesenchymal transition. Clin Cancer Res 2014; 20: 1323–1334. 24443521 10.1158/1078-0432.CCR-13-2429

[17] Paajanen J, Laaksonen S, Ilonen I, *et al*. Clinical features in malignant pleural mesothelioma patients with 5‐year survival and evaluation of original diagnoses. Clin Lung Cancer 2020; 21: e633–e639. 32624414 10.1016/j.cllc.2020.05.020

[18] Berzenji L, Van Schil PE, Carp L. The eighth TNM classification for malignant pleural mesothelioma. Transl Lung Cancer Res 2018; 7: 543–549. 30450292 10.21037/tlcr.2018.07.05 PMC6204412

[19] Öhlund D, Handly‐Santana A, Biffi G, *et al*. Distinct populations of inflammatory fibroblasts and myofibroblasts in pancreatic cancer. J Exp Med 2017; 214: 579–596. 28232471 10.1084/jem.20162024 PMC5339682

[20] Husain AN, Colby TV, Ordóñez NG, *et al*. Guidelines for pathologic diagnosis of malignant mesothelioma: 2017 update of the consensus statement from the International Mesothelioma Interest Group. Arch Pathol Lab Med 2018; 142: 89–108. 28686500 10.5858/arpa.2017-0124-RA

[21] Schindelin J, Arganda‐Carreras I, Frise E, *et al*. Fiji: an open‐source platform for biological‐image analysis. Nat Methods 2012; 9: 676–682. 22743772 10.1038/nmeth.2019 PMC3855844

[22] Berg S, Kutra D, Kroeger T, *et al*. ilastik: interactive machine learning for (bio)image analysis. Nat Methods 2019; 16: 1226–1232. 31570887 10.1038/s41592-019-0582-9

[23] McQuin C, Goodman A, Chernyshev V, *et al*. CellProfiler 3.0: next‐generation image processing for biology. PLoS Biol 2018; 16: e2005970. 29969450 10.1371/journal.pbio.2005970 PMC6029841

[24] Jackson HW, Fischer JR, Zanotelli VRT, *et al*. The single‐cell pathology landscape of breast cancer. Nature 2020; 578: 615–620. 31959985 10.1038/s41586-019-1876-x

[25] Milano MT, Zhang H. Malignant pleural mesothelioma: a population‐based study of survival. J Thorac Oncol 2010; 5: 1841–1848. 20975379 10.1097/JTO.0b013e3181f1cf2b

[26] Valente K, Blackham AU, Levine E, *et al*. A histomorphologic grading system that predicts overall survival in diffuse malignant peritoneal mesothelioma with epithelioid subtype. Am J Surg Pathol 2016; 40: 1243–1248. 27438989 10.1097/PAS.0000000000000696 PMC5029445

[27] Kadota K, Suzuki K, Colovos C, *et al*. A nuclear grading system is a strong predictor of survival in epithelioid diffuse malignant pleural mesothelioma. Mod Pathol 2012; 25: 260–271. 21983936 10.1038/modpathol.2011.146 PMC4080411

[28] Habougit C, Trombert‐Paviot B, Karpathiou G, *et al*. Histopathologic features predict survival in diffuse pleural malignant mesothelioma on pleural biopsies. Virchows Arch 2017; 470: 639–646. 28349237 10.1007/s00428-017-2109-z

[29] Kettunen E, Savukoski S, Salmenkivi K, *et al*. CDKN2A copy number and p16 expression in malignant pleural mesothelioma in relation to asbestos exposure. BMC Cancer 2019; 19: 507. 31138176 10.1186/s12885-019-5652-y PMC6537412

[30] Paulsson J, Ehnman M, Östman A. PDGF receptors in tumor biology: prognostic and predictive potential. Future Oncol 2014; 10: 1695–1708. 25145436 10.2217/fon.14.83

[31] Orimo A, Weinberg RA. Heterogeneity of stromal fibroblasts in tumors. Cancer Biol Ther 2007; 6: 618–619. 18027438 10.4161/cbt.6.4.4255

[32] Frings O, Augsten M, Tobin NP, *et al*. Prognostic significance in breast cancer of a gene signature capturing stromal PDGF signaling. Am J Pathol 2013; 182: 2037–2047. 23583284 10.1016/j.ajpath.2013.02.018

[33] Paulsson J, Sjöblom T, Micke P, *et al*. Prognostic significance of stromal platelet‐derived growth factor β‐receptor expression in human breast cancer. Am J Pathol 2009; 175: 334–341. 19498003 10.2353/ajpath.2009.081030 PMC2708819

[34] Hägglöf C, Hammarsten P, Josefsson A, *et al*. Stromal PDGFRβ expression in prostate tumors and non‐malignant prostate tissue predicts prostate cancer survival. PLoS One 2010; 5: e10747. 20505768 10.1371/journal.pone.0010747 PMC2873980

[35] Attanoos RL, Griffin A, Gibbs AR. The use of immunohistochemistry in distinguishing reactive from neoplastic mesothelium. A novel use for desmin and comparative evaluation with epithelial membrane antigen, p53, platelet‐derived growth factor‐receptor, P‐glycoprotein and Bcl‐2. Histopathology 2003; 43: 231–238. 12940775 10.1046/j.1365-2559.2003.01686.x

[36] Roberts F, Harper CM, Downie I, *et al*. Immunohistochemical analysis still has a limited role in the diagnosis of malignant mesothelioma: a study of thirteen antibodies. Am J Clin Pathol 2001; 116: 253–262. 11488073 10.1309/XL6K-8E62-9FLD-V8Q8

[37] Porta C, Mutti L, Tassi G. Negative results of an Italian Group for Mesothelioma (G.I.Me.) pilot study of single‐agent imatinib mesylate in malignant pleural mesothelioma. Cancer Chemother Pharmacol 2007; 59: 149–150. 16636799 10.1007/s00280-006-0243-4

[38] Ramael M, Buysse C, van den Bossche J, *et al*. Immunoreactivity for the β chain of the platelet‐derived growth factor receptor in malignant mesothelioma and non‐neoplastic mesothelium. J Pathol 1992; 167: 1–4. 1320670 10.1002/path.1711670102

[39] Kothmaier H, Quehenberger F, Halbwedl I, *et al*. EGFR and PDGFR differentially promote growth in malignant epithelioid mesothelioma of short and long term survivors. Thorax 2008; 63: 345–351. 18086752 10.1136/thx.2007.085241

[40] Tsao AS, Lin H, Carter BW, *et al*. Biomarker‐integrated neoadjuvant dasatinib trial in resectable malignant pleural mesothelioma. J Thorac Oncol 2018; 13: 246–257. 29313814 10.1016/j.jtho.2017.10.033

[41] Tsao AS, Harun N, Lee JJ, *et al*. Phase I trial of cisplatin, pemetrexed, and imatinib mesylate in chemonaive patients with unresectable malignant pleural mesothelioma. Clin Lung Cancer 2014; 15: 197–201. 24492162 10.1016/j.cllc.2013.12.008 PMC5080907

[42] Buikhuisen WA, Scharpfenecker M, Griffioen AW, *et al*. A randomized phase II study adding axitinib to pemetrexed‐cisplatin in patients with malignant pleural mesothelioma: a single‐center trial combining clinical and translational outcomes. J Thorac Oncol 2016; 11: 758–768. 26845191 10.1016/j.jtho.2016.01.014

[43] Mathy A, Baas P, Dalesio O, *et al*. Limited efficacy of imatinib mesylate in malignant mesothelioma: a phase II trial. Lung Cancer 2005; 50: 83–86. 15951053 10.1016/j.lungcan.2005.04.010

[44] Zucali PA, Perrino M, De Vincenzo F, *et al*. A phase II study of the combination of gemcitabine and imatinib mesylate in pemetrexed‐pretreated patients with malignant pleural mesothelioma. Lung Cancer 2020; 142: 132–137. 32102735 10.1016/j.lungcan.2020.02.005

[45] Bertino P, Porta C, Barbone D, *et al*. Preliminary data suggestive of a novel translational approach to mesothelioma treatment: imatinib mesylate with gemcitabine or pemetrexed. Thorax 2007; 62: 690–695. 17311837 10.1136/thx.2006.069872 PMC2117287

[46] Kawaguchi K, Murakami H, Taniguchi T, *et al*. Combined inhibition of MET and EGFR suppresses proliferation of malignant mesothelioma cells. Carcinogenesis 2009; 30: 1097–1105. 19380521 10.1093/carcin/bgp097

[47] Rosset EM, Bradshaw AD. SPARC/osteonectin in mineralized tissue. Matrix Biol 2016; 52–54: 78–87. 10.1016/j.matbio.2016.02.001 PMC5327628 26851678

[48] Lansley SM, Pedersen B, Robinson C, *et al*. A subset of malignant mesothelioma tumors retain osteogenic potential. Sci Rep 2016; 6: 36349. 27886205 10.1038/srep36349 PMC5122867

[49] Kao SC, Kirschner MB, Cooper WA, *et al*. A proteomics‐based approach identifies secreted protein acidic and rich in cysteine as a prognostic biomarker in malignant pleural mesothelioma. Br J Cancer 2016; 114: 524–531. 26889976 10.1038/bjc.2015.470 PMC4782201

[50] Zhuo Y, Lin L, Zhang M. Pretreatment thrombocytosis as a significant prognostic factor in malignant mesothelioma: a meta‐analysis. Platelets 2017; 28: 560–566. 27848258 10.1080/09537104.2016.1246712

