# Supplementary material for: High tumor cell platelet‐derived growth factor receptor beta expression is associated with shorter survival in malignant pleural epithelioid mesothelioma
Source: J Pathol Clin Res. 2021 May 6;7(5):482–94. doi: 10.1002/cjp2.218 (PMC8363931; doi:10.1002/cjp2.218)
Supplement: Supplementary file 1 — File S1. Description of staining procedure, antibodies, and imaging [file CJP2-7-482-s005.docx]

**High tumor cell platelet-derived growth factor receptor beta expression is associated with shorter survival in malignant pleural epithelioid mesothelioma**

H Ollila *et al*. *J Pathol Clin Res* DOI: 10.1002/cjp2.218

**Supplementary material, File S1.** Description of staining procedure, antibodies and imaging.

Reference numbers refer to the list in the main paper.

**Staining procedure and antibodies**

For panel 1, the first-round staining primary antibodies and detection reagents were the following: PDGFRB (1:100, Cell Signaling Technology #3169) with TSA-488 detection, PDGFRA (1:50, Cell Signaling Technology #5241) with TSA-555 detection, aSMA (1:200, Agilent Dako M0851) with anti-mouse-AF647 detection, FAP (1:500, Abcam ab207178) with TSA-750 detection. Prior to the second-round staining, slides were stained with DAPI (Roche, 1.6 µg/ml), mounted with ProLong Gold (Thermo Fisher Scientific), whole-slide imaged (see imaging below) and coverslips removed. The second-round staining consisted of CK5 antibody (1:400; Abcam ab52635) with anti-rabbit-AF647 detection and re-stain of DAPI.

For panel 2, only one round of staining was performed due to smaller number of included antibodies. The primary antibodies and detection reagents were the following: SPARC (1:500, R&D Systems AF941) with TSA-488 detection, Collagen I (1:200, Abcam ab34710) with TSA-555 detection, POSTN (1:500, Abcam ab215199) with AF647 detection, anti-mesothelioma cocktail consisting of several antibodies (Calretinin, 1:100, Agilent Dako M724529-2; CK5/6, 1:50, Cell Marque 356M-15; CK5, 1:100, Abcam ab17130) with TSA-750 detection.

Mesothelioma cancer cells were stained using CK5 in panel 1 and CK5, CK5&6 and Calretinin antibody cocktail in panel 2. CK5, CK5&6 and Calretinin are all shown to be reliable markers for detecting the MPM cells [15,20]. The Spearman’s rank correlation coefficient was 0.78 (p<0.001) between these two mesothelioma cancer cell antibody stainings. As a reference, the Spearman’s rho between the nuclei (DAPI) stainings in these two panels was 0.82 (p<0.001). Thus, these two mesothelial stainings are comparable.

**Imaging**

The whole-slide TMA imaging (in order to achieve 5-channel fluorescence images) was implemented using Axio Scan.Z1 Digital Slide Scanner (The Zeiss™, Germany) equipped with 20X (0.8NA) Plan-Apochromat objective (The Zeiss™, Germany), ORCA-Flash 4.0 V2 Digital CMOS camera (Hamamatsu Photonics K.K., Japan) and Colibri.7 LED light source (The Zeiss™, Germany). DAPI, FITC, Cy3, Cy5 and Cy7 filters were used. The exposure times in panel 1 were the following: DAPI = 1.1 ms, FITC (PDGFRB) = 11.2 ms, Cy3 (PDGFRA) = 50 ms, Cy5 (aSMA) = 10.9 ms, Cy7 (FAP) = 82 ms, second round Cy5 (CK5) = 40 ms and DAPI = 4 ms. Respectively in panel 2: DAPI = 2 ms, FITC (SPARC) = 1.5 ms, Cy3 (Collagen I) = 5 ms, Cy5 (POSTN) = 50 ms, Cy7 (anti-mesothelioma cocktail) = 300 ms.
