# Supplementary material for: High tumor cell platelet‐derived growth factor receptor beta expression is associated with shorter survival in malignant pleural epithelioid mesothelioma
Source: J Pathol Clin Res. 2021 May 6;7(5):482–94. doi: 10.1002/cjp2.218 (PMC8363931; doi:10.1002/cjp2.218)
Supplement: Supplementary file 2 — File S2. Detailed information about digital pixel‐based image analysis and quality control of the TMA spots [file CJP2-7-482-s002.docx]

**High tumor cell platelet-derived growth factor receptor beta expression is associated with shorter survival in malignant pleural epithelioid mesothelioma**

H Ollila *et al*. *J Pathol Clin Res* DOI: 10.1002/cjp2.218

**Supplementary material, File S2.** Detailed information about digital pixel-based image analysis and quality control of the TMA spots.

Reference numbers refer to the list in the main paper.

**Detailed information about digital pixel-based image analysis**

Following the whole-slide TMA imaging, the 5-channel fluorescence images were exported as single-channel grayscale images (64 Bit, Big Tiff Format) and resized to a quarter of the original image size. Further, the images were cropped to individual TMA spots using Fiji (version 2.0 for Mac OS X)[21]. The TMA spot images from the second staining round were registered (overlaid) with the first-round images using nuclei (DAPI) stainings from both staining rounds. Registering was performed using MATLAB and Statistics Toolbox Release 2018b (The MathWorks Inc., Natick, Massachusetts, US).

Ilastik (version 1.3.3post1 for MacOS)[22] was used to mask auto fluorescence (e.g. red blood cells), blood vessels, tissue (all other signal in the image excluding the features described before) and empty (background) from the images.

The final image analysis pipeline was performed using CellProfiler (version 3.1.9)[23]. At first, the masked tissue was further classified into different tissue components (mesothelioma, total stroma and different stromal components). The mesothelioma component was defined by tumor cell marker signal (CK5 in panel 1 and CK5, CK5/6 and calretinin marker combination in panel 2) and the remaining tissue area was defined as stroma. Further, the stroma was classified into different stromal components (meso zones 1–4 and vessel zones 1–4). The meso zones 1 to 4 goes from proximal to distal, proximal being closest to the tumor border. Starting from the tumor border (being pixel 0), zone 1 extends from pixel 1 to 10, zone 2 from pixel 11 to 20, zone 3 from pixel 21 to 30 and zone for from pixel 31 onwards. One pixel is 1.2 µm wide. The stromal areas around the vessels (vessel zones 1–4) were defined similarly in panel 1. Finally, the mean intensity (also referred to as “expression” in the text) of each single channel was measured in these tissue components. The average of the mean intensities between the parallel TMA spots per patient was calculated.

The image analysis pipeline files are available upon request.

**Quality control of the TMA spots**

The digital pixel-based image analyses of panels 1 and 2 included 70 and 72 patients, respectively, due to quality control of the TMA spots prior to analyses. Only the TMA spots representing tumor tissue were included in analyses. Folded or broken spots were excluded as well as the spots not including tumor cells. The quality check and the presence of the tumor cells was evaluated by screening the scanned hematoxylin and eosin (MIM and HO) and fluorescence stained slides (HO).
