# Supplementary material for: High tumor cell platelet‐derived growth factor receptor beta expression is associated with shorter survival in malignant pleural epithelioid mesothelioma
Source: J Pathol Clin Res. 2021 May 6;7(5):482–94. doi: 10.1002/cjp2.218 (PMC8363931; doi:10.1002/cjp2.218)
Supplement: Supplementary file 3 — File S3. Primary cohort post hoc association analyses [file CJP2-7-482-s001.docx]

**High tumor cell platelet-derived growth factor receptor beta expression is associated with shorter survival in malignant pleural epithelioid mesothelioma**

H Ollila *et al*. *J Pathol Clin Res* DOI: 10.1002/cjp2.218

**Supplementary material, File S3.** Primary cohort *post-hoc* association analyses.

**Variable definitions**

The tumor size was defined as tumoral extent multiplied by maximal tumoral thickness. Mitoses were defined as 1 if the average over 10 high power fields was 1.40 or less, as 2 if the average over 10 high power fields was 1.50–4.40 and as 3 if the average over 10 high power fields was 4.50 or more. Nuclear atypia was classified into three groups: mild, moderate, and severe. Further, nuclear grading was finally defined as a sum of mitoses and nuclear atypia. The tumor architecture was categorized as favorable if the most predominant growth pattern was either trabecular or tubulopapillary and as unfavorable if the most predominant growth pattern was either solid or micropapillary. BAP1 tumor status was examined by performing BAP1 DAB staining and further analyzing the BAP1 positive tumors by visually scoring the scanned images. We were able to determine the status for 71 of 74 patients in our primary study cohort. The scoring was performed by HO, MIM and TP.

| **Table 1.** Association analyses and Univariate Cox regression. Primary cohort. | | | | | |
| --- | --- | --- | --- | --- | --- |
| **Tumor cell PDGFRB mean intensity association analyses** | | | **Univariate Cox regression** | | |
| **Variable** | **Definition** | **Result** |  |  |  |
|  |  |  | ***p*-value** | **HR** | ***p* corr.** |
| Tumor size | Tumor extent (0–3) x maximum tumor thickness (mm). | 0.103 (*p*=0.424) | 0.715 | 1.00 | 1.00 |
| Clinical stage | Categories 1–4. Defined according to 8th edition of the TNM. | *p*=0.579 | 0.220 | 1.14 | 1.00 |
| Nuclear grading | Categories 2–6. Sum of mitoses (1–3) and nuclear atypia (1–3). | *p*=0.347 | 0.001*** | 1.95 | 0.001*** |
| Tumor architecture | Categories 1 and 2. 1=Favorable (trabecular or tubulopapillary), 2=Unfavorable (solid or micropapillary). | *p*=0.020* | 0.001*** | 2.69 | 0.001*** |
| Mitoses | Average mitotic count per 10 HPF. | -0.02 (*p*=0.890) | 0.003** | 1.12 | 0.017* |
| BAP1 | Samples were dichotomized according to tumor cell BAP1 positivity. | *p*=0.473 | 0.030* | 1.80 | 0.030* |
| A HR >1 indicates an increased risk of death and HR <1 indicates a decreased risk of death.  **p*<0.05, ***p*<0.01, ****p*<0.001.  Tumor size analyzed using the Spearman’s rho Test.  Clinical stage analyzed using the Kruskal-Wallis Test.  Nuclear grading analyzed using the Kruskal-Wallis Test.  Tumor architecture analyzed using the Mann-Whitney U Test.  Mitoses analyzed using the Spearman’s rho Test.  BAP1 analyzed using the Mann-Whitney U Test.  Abbreviations: HR, hazard ratio; *p* corr, Bonferroni corrected *p*-value; TNM, tumor, lymph nodes, metastasis; HPF, high-power field. | | | | | |

**Figure 1.** Showing tumor cell PDGFRB mean intensity distribution according to tumor architecture in primary cohort. The tumor cell PDGFRB mean intensity was higher in the unfavorable tumor architecture group. The groups were compared by using the Mann-Whitney U test.


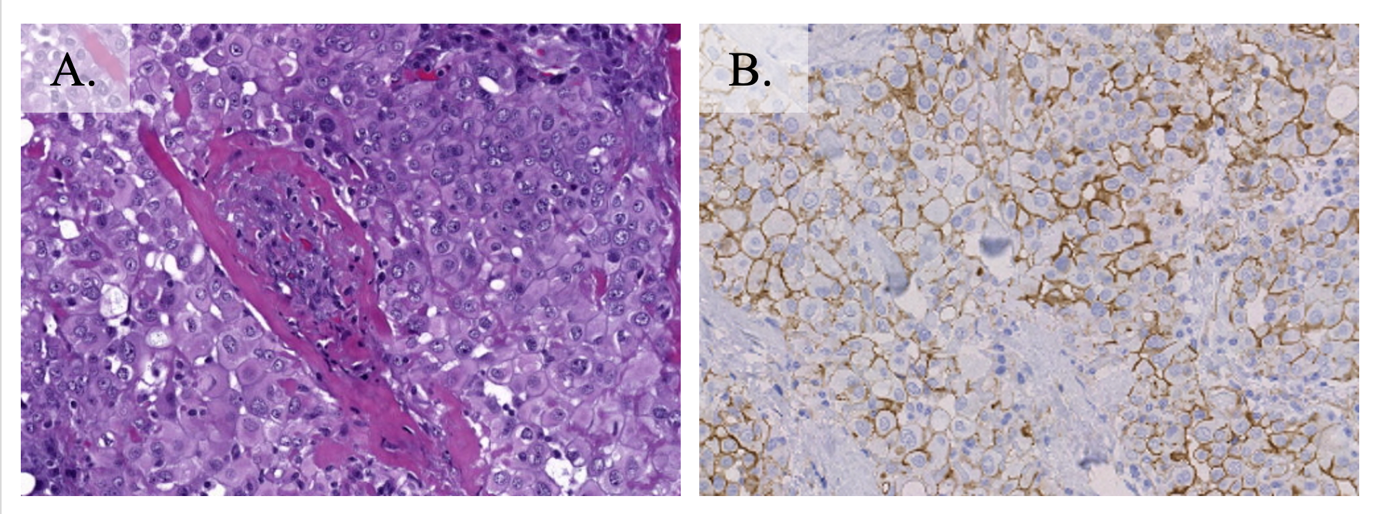


**Figure 2.** Image showing a patient with solid tumor architecture (A.) (H&E staining) and high tumor cell PDGFRB expression (B.) (PDGFRB DAB staining).

| **Table 2.** Multivariable Cox regression analysis. Primary cohort (n=59). | | |
| --- | --- | --- |
| **Variable** | **HR (95 % CI)** | ***p*-value** |
| Age | 1.01 (0.98–1.04) | 0.430 |
| Sex |  |  |
| Male | 1.0 |  |
| Female | 0.47 (0.19–1.16) | 0.100 |
| Side of the disease |  |  |
| Right | 1.0 |  |
| Left | 0.77 (0.40–1.47) | 0.428 |
| Clinical stage |  |  |
| Low | 1.0 |  |
| High | 1.54 (0.83–2.88) | 0.174 |
| Histology |  |  |
| Epithelioid | 1.0 |  |
| Biphasic | 1.06 (0.23–4.92) | 0.939 |
| Nuclear grading | 1.07 (0.60–1.90) | 0.819 |
| Tumor architecture |  |  |
| Favorable | 1.0 |  |
| Unfavorable | 3.20 (1.32–7.76) | 0.010** |
| Mitoses | 1.07 (0.97–1.18) | 0.164 |
| BAP1 |  |  |
| Negative | 1.0 |  |
| Positive | 1.60 (0.72–3.55) | 0.250 |
| Tumor cell PDGFRB mean intensity | 1.02 (1.00–1.03) | 0.006** |
| A HR >1 indicates an increased risk of death and a HR <1 indicates a decreased risk of death.  **p*<0.05, ***p*<0.01, ****p*<0.001.  The model fulfilled the proportional hazard assumption.  Abbreviations: CI, confidence interval; HR, hazard ratio; PDGFRB, Platelet Derived Growth Factor Receptor Beta. | | |

| **Table 3.** Univariate Cox regression analysis separately for tumor architecture groups. Primary cohort. | | |
| --- | --- | --- |
| **Variable** | **HR** | ***p*-value** |
| **Favorable tumor architecture (predominantly trabecular or tubulopapillary growth pattern) (n=27)** | | |
| Clinical stage | 1.07 | 0.68 |
| Tumor size | 1.00 | 0.42 |
| Mitoses | 1.08 | 0.37 |
| Nuclear grading | 1.21 | 0.55 |
| Tumor cell PDGFRB mean intensity | 1.03 | 0.03* |
| **Unfavorable tumor architecture (predominantly solid or micropapillary growth pattern) (n=39)** | | |
| Clinical stage | 1.05 | 0.72 |
| Tumor size | 1.00 | 0.61 |
| Mitoses | 1.14 | 0.008** |
| Nuclear grading | 1.76 | 0.02* |
| Tumor cell PDGFRB mean intensity | 1.01 | 0.04* |
| A HR >1 indicates an increased risk of death and a HR <1 indicates a decreased risk of death.  **p*<0.05, ***p*<0.01, ****p*<0.001.  Abbreviations: HR, hazard ratio; PDGFRB, Platelet Derived Growth Factor Receptor Beta. | | |
