# Supplementary material for: High tumor cell platelet‐derived growth factor receptor beta expression is associated with shorter survival in malignant pleural epithelioid mesothelioma
Source: J Pathol Clin Res. 2021 May 6;7(5):482–94. doi: 10.1002/cjp2.218 (PMC8363931; doi:10.1002/cjp2.218)
Supplement: Supplementary file 4 — File S4. External validation [file CJP2-7-482-s003.docx]

**High tumor cell platelet-derived growth factor receptor beta expression is associated with shorter survival in malignant pleural epithelioid mesothelioma**

H Ollila *et al*. *J Pathol Clin Res* DOI: 10.1002/cjp2.218

**Supplementary material, File S4.** External validation.

| **Table 1.** Patient characteristics, validation cohort. | |
| --- | --- |
| **Variable** | **All patients (n=117)** |
| **Months to death from diagnosis***, median (IQR) | 10 (­2.0–17) |
| **Age** (years) at time of diagnosis, median (IQR) | 63 (55–73) |
| **Sex** |  |
| Female | 13 (11%) |
| Male | 104 (89%) |
| **Histology** |  |
| Epithelioid | 76 (65%) |
| Sarcomatoid | 22 (19%) |
| Mixed (biphasic) | 19 (16%) |
| *1 patient was still alive at end of follow up (Dec 14, 2020) and was the only patient living longer than 60 months.  Abbreviations: IQR, Interquartile Range | |

**
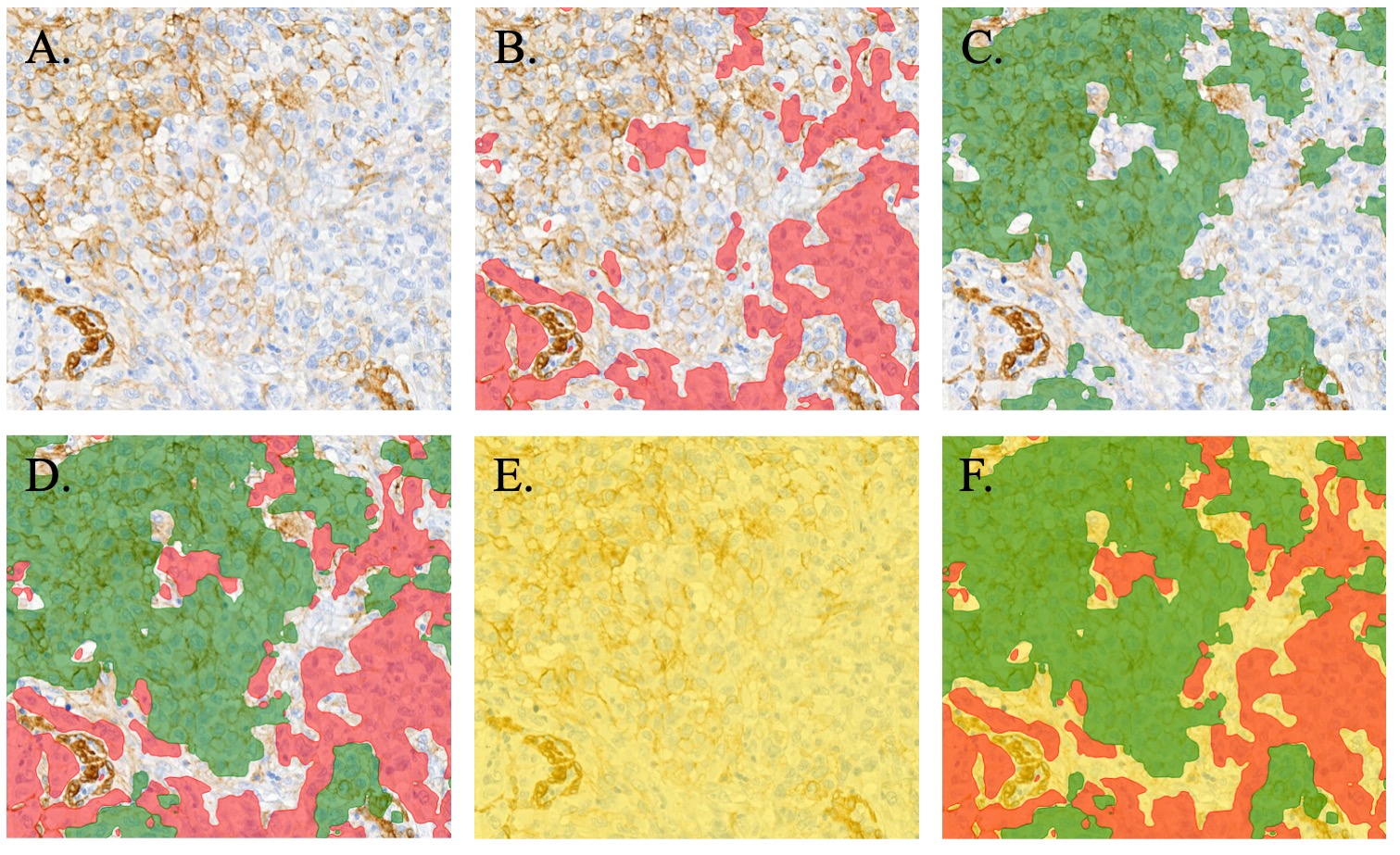
**

**Figure 1. Trained AI-model.** A. PDGFRB DAB staining. B. PDGFRB-negative mesothelioma tumor cells in red. C. PDGFRB-positive mesothelioma tumor cells in green. D. Combination of B. and C. E. Tissue. F. Combination of D and E.

| **Table 2.** Univariate Cox regression analysis. Validation cohort. | | | |
| --- | --- | --- | --- |
| **Variable (n=117)** | **HR** | ***p*-value** | ***p* corr.** |
| Tumor PDGFRB area in proportion to total tumor area | 2.10 | 0.010** | 0.041* |
| A HR >1 indicates an increased risk of death and a HR <1 indicates a decreased risk of death.  **p*<0.05, ***p*<0.01, ****p*<0.001.  Abbreviations: HR, hazard ratio; PDGFRB, Platelet Derived Growth Factor Receptor Beta; *p* corr., Bonferroni corrected *p*-value. | | | |

| **Table 3.** Multivariable Cox regression analysis. Validation cohort. | | |
| --- | --- | --- |
| **Variable (n=117)** | **HR (95% CI)** | ***p*-value** |
| Age | 1.01 (0.99–1.03) | 0.208 |
| Female sex | 0.65 (0.36–1.17) | 0.151 |
| Histology |  |  |
| Epithelioid | 1.0 |  |
| Biphasic | 1.51 (0.86–2.66) | 0.154 |
| Sarcomatoid | 5.24 (2.79–9.85) | <0.001*** |
| Tumor PDGFRB area in proportion to total tumor area | 1.25 (0.66–2.35) | 0.489 |
| A HR >1 indicates an increased risk of death and a HR <1 indicates a decreased risk of death.  **p*<0.05, ***p*<0.01, ****p*<0.001.  The model fulfilled the proportional hazard assumption.  Abbreviations: CI, confidence interval; HR, hazard ratio; PDGFRB, Platelet Derived Growth Factor Receptor Beta | | |

| **Table 4.** Multivariable Cox regression analysis. Validation cohort, only epithelioid mesotheliomas. | | |
| --- | --- | --- |
| **Variable (n=76)** | **HR (95% CI)** | ***p*-value** |
| Age | 1.01 (0.98–1.03) | 0.668 |
| Female sex | 0.70 (0.32–1.57) | 0.389 |
| Tumor PDGFRB area in proportion to total tumor area | 2.13 (0.84–5.40) | 0.112 |
| Multivariable Cox regression adjusted for age and sex. A HR >1 indicates an increased risk of death and a HR <1 indicates a decreased risk of death.  **p*<0.05, ***p*<0.01, ****p*<0.001.  The model fulfilled the proportional hazard assumption.  Abbreviations: CI, confidence interval; HR, hazard ratio; PDGFRB, Platelet Derived Growth Factor Receptor Beta | | |


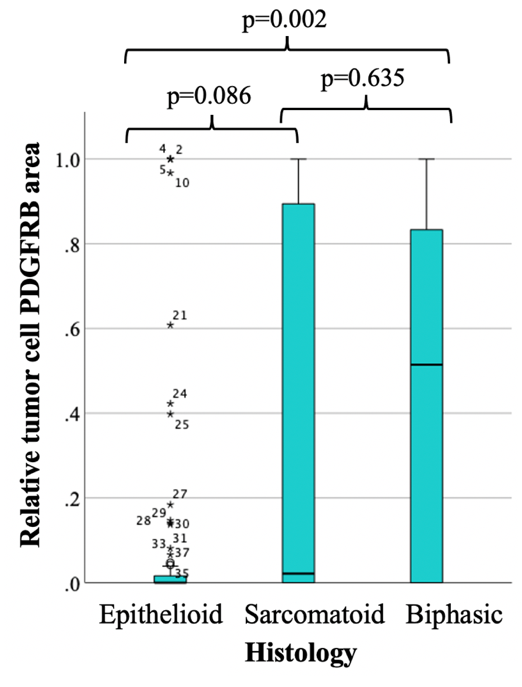


**Figure 2.** Showing relative tumor cell PDGFRB area distribution according to histology in validation cohort. The groups were compared by using the Mann Whitney U test.
