# Supplementary material for: High tumor cell platelet‐derived growth factor receptor beta expression is associated with shorter survival in malignant pleural epithelioid mesothelioma
Source: J Pathol Clin Res. 2021 May 6;7(5):482–94. doi: 10.1002/cjp2.218 (PMC8363931; doi:10.1002/cjp2.218)
Supplement: Supplementary file 5 — File S5. Marker mean intensity distributions in mesothelial and stromal components, meso zones 1–4 and vessel zones 1–4 [file CJP2-7-482-s004.docx]

**High tumor cell platelet-derived growth factor receptor beta expression is associated with shorter survival in malignant pleural epithelioid mesothelioma**

H Ollila *et al*. *J Pathol Clin Res* DOI: 10.1002/cjp2.218

**Supplementary material, File S5.** Marker mean intensity distributions in A) mesothelial and stromal components, B) meso zones 1–4, C) vessel zones 1–4.
