# Supplementary material for: High tumor cell platelet‐derived growth factor receptor beta expression is associated with shorter survival in malignant pleural epithelioid mesothelioma
Source: J Pathol Clin Res. 2021 May 6;7(5):482–94. doi: 10.1002/cjp2.218 (PMC8363931; doi:10.1002/cjp2.218)
Supplement: Supplementary file 6 — File S6. Biphasic mesotheliomas in the primary cohort [file CJP2-7-482-s007.docx]

**High tumor cell platelet-derived growth factor receptor beta expression is associated with shorter survival in malignant pleural epithelioid mesothelioma**

H Ollila *et al*. *J Pathol Clin Res* DOI: 10.1002/cjp2.218

**Supplementary material, File S6.** Biphasic mesotheliomas (n=5) in the primary cohort.

We analyzed the differences between the expression (mean intensity) of the fibroblast markers in epithelioid and sarcomatoid tumor cell areas (meso) and the stromal areas surrounding them (stroma) in biphasic tumors.

1. Panel 1


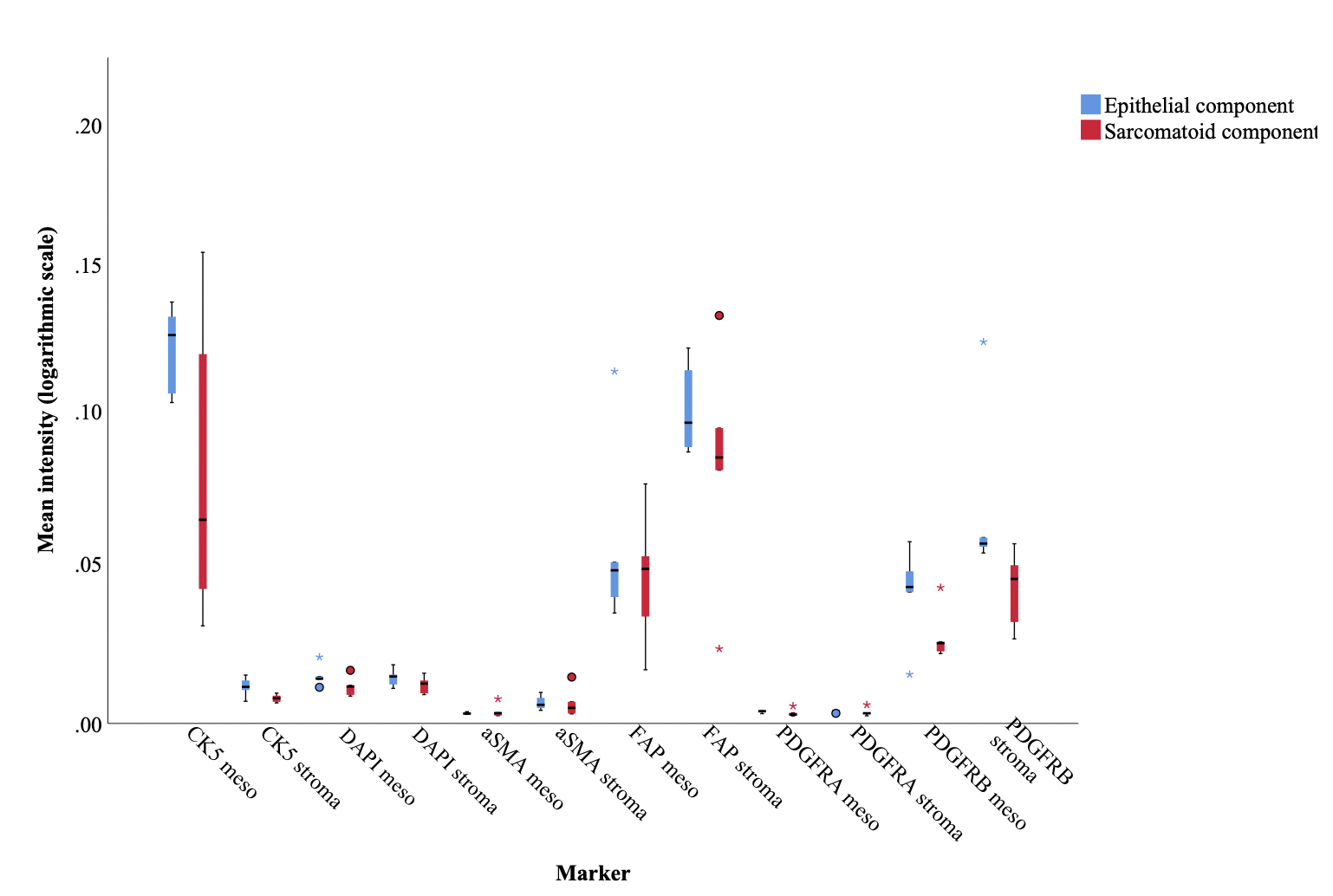


1. Panel 2


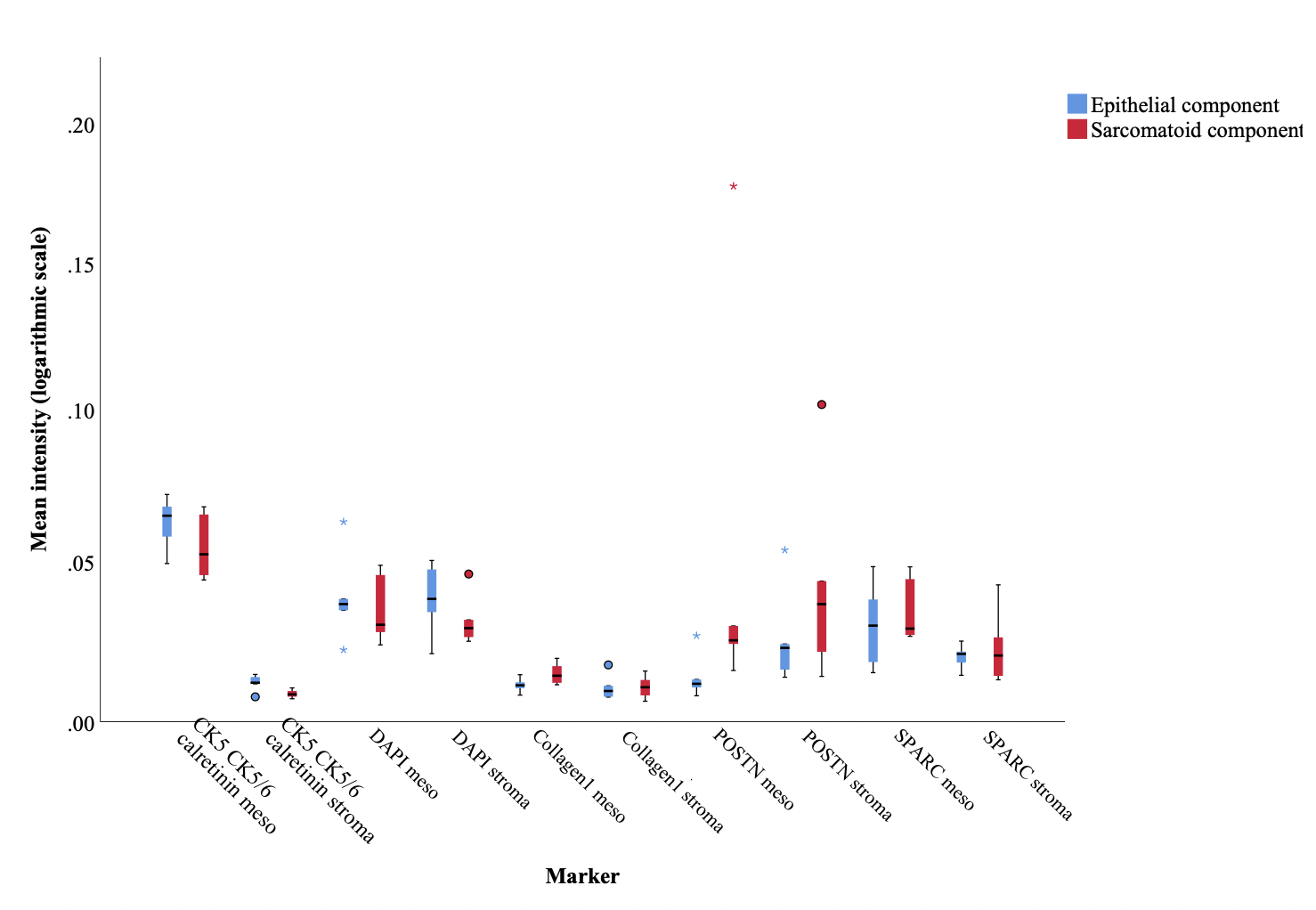


Statistically significant differences between the epithelial and sarcomatoid components were studied using the Mann-Whitney U test:

| **Marker** | ***p*-value** |
| --- | --- |
| CK5 meso | 0.310 |
| CK5 stroma | 0.056 |
| DAPI meso | 0.222 |
| DAPI stroma | 0.421 |
| PDGFRA meso | 0.222 |
| PDGFRA stroma | 0.310 |
| PDGFRB meso | 0.222 |
| PDGFRB stroma | 0.032* |
| FAP meso | 0.841 |
| FAP stroma | 0.310 |
| aSMA meso | 0.841 |
| aSMA stroma | 0.690 |
| CK5+CK5/6+calretinin meso | 0.310 |
| CK5+CK5/6+calretinin stroma | 0.095 |
| DAPI meso | 0.841 |
| DAPI stroma | 0.310 |
| SPARC meso | 0.841 |
| SPARC stroma | 1.00 |
| Collagen1 meso | 0.151 |
| Collagen1 stroma | 1.00 |
| POSTN meso | 0.056 |
| POSTN stroma | 0.584 |
| **p*<0.05, ***p*<0.01, ****p*<0.001.  Abbreviations: meso, mesothelial tumor cells; CK5, cytokeratin 5; CK5/6, cytokeratin 5/6; FAP, fibroblast activation protein; PDGFRA, platelet derived growth factor receptor alpha; PDGFRB, platelet derived growth factor receptor beta; aSMA, alpha smooth muscle actin; SPARC, secreted protein acidic and rich in cysteine; POSTN, periostin. | |
