# Supplementary material for: High tumor cell platelet‐derived growth factor receptor beta expression is associated with shorter survival in malignant pleural epithelioid mesothelioma
Source: J Pathol Clin Res. 2021 May 6;7(5):482–94. doi: 10.1002/cjp2.218 (PMC8363931; doi:10.1002/cjp2.218)
Supplement: Supplementary file 7 — File S7. Univariate Cox regression analysis showing the association between individual markers and survival [file CJP2-7-482-s006.docx]

**High tumor cell platelet-derived growth factor receptor beta expression is associated with shorter survival in malignant pleural epithelioid mesothelioma**

H Ollila *et al*. *J Pathol Clin Res* DOI: 10.1002/cjp2.218

**Supplementary material, File S7.** Univariate Cox regression analysis showing the association between individual markers and survival.

| **mfIHC variable** | ***p*** | **HR** | ***p* corr** |
| --- | --- | --- | --- |
| **Panel 1** | | | |
| CK5_meso | ﻿0.62 | ﻿1.00 | ﻿1.00 |
| CK5_stroma | ﻿0.57 | ﻿1.02 | ﻿1.00 |
| CK5_meso_Z1 | 0.44 | 1.03 | 1.00 |
| CK5_meso_Z2 | 0.07 | 1.17 | 1.00 |
| CK5_meso_Z3 | 0.07 | 1.20 | 1.00 |
| CK5_meso_Z4 | 0.10 | 1.15 | 1.00 |
| CK5_vessel_Z1 | 0.33 | 1.04 | 1.00 |
| CK5_vessel_Z2 | 0.32 | 1.03 | 1.00 |
| CK5_vessel_Z3 | 0.39 | 1.03 | 1.00 |
| CK5_vessel_Z4 | 0.45 | 1.03 | 1.00 |
| DAPI_meso | ﻿0.46 | ﻿1.01 | ﻿1.00 |
| DAPI_stroma | ﻿0.03* | ﻿1.02 | ﻿1.00 |
| DAPI_meso_Z1 | 0.01* | 1.03 | 0.79 |
| DAPI_meso_Z2 | 0.02* | 1.03 | 1.00 |
| DAPI_meso_Z3 | 0.02* | 1.02 | 1.00 |
| DAPI_meso_Z4 | 0.13 | 1.01 | 1.00 |
| DAPI_vessel_Z1 | 0.11 | 1.02 | 1.00 |
| DAPI_vessel_Z2 | 0.03* | 1.03 | 1.00 |
| DAPI_vessel_Z3 | 0.01* | 1.03 | 0.77 |
| DAPI_vessel_Z4 | 0.02* | 1.03 | 1.00 |
| FAP_meso | ﻿0.08 | ﻿1.01 | ﻿1.00 |
| FAP_stroma | ﻿0.03* | ﻿1.01 | ﻿1.00 |
| FAP_meso_Z1 | 0.04* | 1.01 | 1.00 |
| FAP_meso_Z2 | 0.03* | 1.01 | 1.00 |
| FAP_meso_Z3 | 0.04* | 1.01 | 1.00 |
| FAP_meso_Z4 | 0.02* | 1.01 | 1.00 |
| FAP_vessel_Z1 | 0.05 | 1.00 | 1.00 |
| FAP_vessel_Z2 | 0.04* | 1.00 | 1.00 |
| FAP_vessel_Z3 | 0.02* | 1.01 | 1.00 |
| FAP_vessel_Z4 | 0.05 | 1.01 | 1.00 |
| PDGFRA_meso | ﻿0.49 | ﻿1.02 | ﻿1.00 |
| PDGFRA_stroma | ﻿0.33 | ﻿1.03 | ﻿1.00 |
| PDGFRA_meso_Z1 | 0.49 | 1.02 | 1.00 |
| PDGFRA_meso_Z2 | 0.30 | 1.04 | 1.00 |
| PDGFRA_meso_Z3 | 0.37 | 1.03 | 1.00 |
| PDGFRA_meso_Z4 | 0.39 | 1.03 | 1.00 |
| PDGFRA_vessel_Z1 | 0.28 | 1.04 | 1.00 |
| PDGFRA_vessel_Z2 | 0.30 | 1.04 | 1.00 |
| PDGFRA_vessel_Z3 | 0.36 | 1.03 | 1.00 |
| PDGFRA_vessel_Z4 | 0.41 | 1.03 | 1.00 |
| PDGFRB_meso | ﻿0.0006*** | ﻿1.02 | ﻿0.04* |
| PDGFRB_stroma | ﻿0.14 | ﻿1.01 | ﻿1.00 |
| PDGFRB_meso_Z1 | 0.17 | 1.01 | 1.00 |
| PDGFRB_meso_Z2 | 0.32 | 1.00 | 1.00 |
| PDGFRB_meso_Z3 | 0.25 | 1.00 | 1.00 |
| PDGFRB_meso_Z4 | 0.10 | 1.01 | 1.00 |
| **mfIHC variable** | ***p*** | **HR** | ***p* corr** |
| **Panel 1 (cont.)** | | | |
| PDGFRB_vessel_Z1 | 0.68 | 1.00 | 1.00 |
| PDGFRB_vessel_Z2 | 0.15 | 1.01 | 1.00 |
| PDGFRB_vessel_Z3 | 0.19 | 1.01 | 1.00 |
| PDGFRB_vessel_Z4 | 0.32 | 1.01 | 1.00 |
| aSMA_meso | ﻿0.19 | ﻿1.09 | ﻿1.00 |
| aSMA_stroma | ﻿0.35 | ﻿1.04 | ﻿1.00 |
| aSMA_meso_Z1 | 0.31 | 1.04 | 1.00 |
| aSMA_meso_Z2 | 0.27 | 1.04 | 1.00 |
| aSMA_meso_Z3 | 0.29 | 1.04 | 1.00 |
| aSMA_meso_Z4 | 0.39 | 1.02 | 1.00 |
| aSMA_vessel_Z1 | 0.84 | 1.00 | 1.00 |
| aSMA_vessel_Z2 | 0.53 | 1.02 | 1.00 |
| aSMA_vessel_Z3 | 0.54 | 1.02 | 1.00 |
| aSMA_vessel_Z4 | 0.74 | 1.01 | 1.00 |
| **Panel 2** | | | |
| Calretinin+CK5/6+CK5_meso | 0.70 | 1.00 | ﻿1.00 |
| Calretinin+CK5/6+CK5_stroma | 0.68 | 1.02 | ﻿1.00 |
| Calretinin+CK5/6+CK5_meso_Z1 | 0.32 | 1.02 | ﻿1.00 |
| Calretinin+CK5/6+CK5_meso_Z2 | ﻿0.37 | ﻿1.06 | ﻿1.00 |
| Calretinin+CK5/6+CK5_meso_Z3 | ﻿0.63 | ﻿1.05 | ﻿1.00 |
| Calretinin+CK5/6+CK5_meso_Z4 | ﻿0.89 | ﻿0.99 | ﻿1.00 |
| DAPI_meso | ﻿0.42 | ﻿1.00 | ﻿1.00 |
| DAPI_stroma | ﻿0.10 | 1.01 | ﻿1.00 |
| DAPI_meso_Z1 | ﻿0.10 | ﻿1.01 | ﻿1.00 |
| DAPI_meso_Z2 | ﻿0.06 | ﻿1.01 | ﻿1.00 |
| DAPI_meso_Z3 | ﻿0.11 | ﻿1.01 | ﻿1.00 |
| DAPI_meso_Z4 | ﻿0.53 | ﻿1.00 | ﻿1.00 |
| SPARC_meso | ﻿0.17 | ﻿1.02 | ﻿1.00 |
| SPARC_stroma | 0.006** | ﻿1.07 | ﻿0.21 |
| SPARC_meso_Z1 | ﻿0.005** | ﻿1.06 | ﻿0.16 |
| SPARC_meso_Z2 | ﻿0.002** | ﻿1.07 | ﻿0.08 |
| SPARC_meso_Z3 | 0.003** | ﻿1.07 | ﻿0.11 |
| SPARC_meso_Z4 | ﻿0.007** | ﻿1.07 | ﻿0.26 |
| POSTN_meso | ﻿0.33 | ﻿1.01 | ﻿1.00 |
| POSTN_stroma | ﻿0.23 | ﻿1.00 | ﻿1.00 |
| POSTN_meso_Z1 | ﻿0.31 | ﻿1.00 | ﻿1.00 |
| POSTN_meso_Z2 | ﻿0.28 | ﻿1.00 | ﻿1.00 |
| POSTN_meso_Z3 | ﻿0.21 | ﻿1.00 | ﻿1.00 |
| POSTN_meso_Z4 | ﻿0.16 | ﻿1.00 | ﻿1.00 |
| Collagen1_meso | ﻿0.46 | ﻿0.97 | ﻿1.00 |
| Collagen1_stroma | ﻿0.36 | ﻿1.05 | ﻿1.00 |
| Collagen1_meso_Z1 | ﻿0.71 | ﻿1.02 | ﻿1.00 |
| Collagen1_meso_Z2 | ﻿0.32 | ﻿1.04 | ﻿1.00 |
| Collagen1_meso_Z3 | ﻿0.32 | ﻿1.04 | ﻿1.00 |
| Collagen1_meso_Z4 | ﻿0.76 | ﻿1.01 | ﻿1.00 |
| A HR > 1 indicates an increased risk for death and a HR < 1 indicates a decreased risk for death.  *p* stands for *p*-value, HR for hazard ratio, *p* corr for Bonferroni corrected *p*-value. mfIHC variables as average mean intensities in mesothelial and stromal tissue components. Average mean intensities multiplied by 1,000. **p*<0.05, ***p*<0.01, ****p*<0.001.  Abbreviations: mfIHC, multiplexed fluorescence immunohistochemistry; meso, mesothelioma; CK5, cytokeratin 5; CK5/6, cytokeratin 5/6; Z1–Z4, Zone 1–Zone 4; FAP, fibroblast activation protein; PDGFRA, platelet derived growth factor receptor alpha; PDGFRB, platelet derived growth factor receptor beta; aSMA, alpha smooth muscle actin; SPARC, secreted protein acidic and rich in cysteine; POSTN, periostin. | | | |
